# Multiple drilling is not effective in reducing the rate of conversion to Total hip Arthroplasty in early-stage nontraumatic osteonecrosis of the femoral head: a case-control comparative study with a natural course

**DOI:** 10.1186/s12891-021-04418-y

**Published:** 2021-06-12

**Authors:** Zunhan Liu, Xuetao Yang, Yuhan Li, Wei-Nan Zeng, Enze Zhao, Zongke Zhou

**Affiliations:** Department of Orthopedic Surgery, West China Hospital, West China Medical School, Sichuan University, #37 Guoxue Road, Chengdu, Sichuan Province 610041 People’s Republic of China

**Keywords:** Multiple drilling, Risk factors, Total hip arthroplasty, Autogenous bone grafting, Osteoarthritis

## Abstract

**Background:**

To determine whether multiple drilling is effective in postponing the need for total hip arthroplasty (THA) in early-stage nontraumatic osteonecrosis of the femoral head (ONFH).

**Methods:**

We identified 514 patients who were diagnosed with early-stage ONFH between January 2008 and December 2018. One hundred ninety-six patients underwent multiple drilling, and 318 patients had a natural course of progression. One hundred fifty-nine patients were selected for each group after case-control matching for preoperative demographics and modified Ficat and Arlet stage. The rates of THA conversion were compared. We also performed Cox regression to identify risk factors associated with THA conversion in patients who underwent multiple drilling.

**Results:**

Kaplan-Meier survivorship with an endpoint of THA for nontraumatic reasons were not significantly different between the multiple drilling group (75.6, 95% confidence interval 67.8–83.4%) and the natural course group (72.2, 95% confidence interval 64.8–79.6%) at 5 years (log-rank, *P* = .191). In the Cox regression model, a larger extent of necrotic lesion, bone marrow edema (BME), and higher postoperative work intensity significantly increased the risk of THA conversion (*P* < .05). Among patients treated with autogenous bone grafting, there was a lower risk of failure in patients with necrotic lesion less than 15% (*P* < .05).

**Conclusions:**

Multiple drilling is not effective in reducing the rate of THA conversion in early-stage nontraumatic ONFH. The risk of conversion to THA after multiple drilling is increased by a larger extent of necrotic lesion, presence of BME, and higher postoperative work intensity in patients with early-stage ONFH.

**Trial registration:**

The trial was registered in the Chinese Clinical Trial Registry (ChiCTR2000035180) dated 2 August 2020.

## Background

Nontraumatic osteonecrosis of the femoral head (ONFH) is a clinical entity originating from impaired circulation of femoral head terminal blood flow, if not treated effectively, leading to femoral head collapse and, ultimately, arthritis of the hips. Total hip arthroplasty (THA) is one of the most successful health care interventions for patients with postcollapse ONFH [1]. However, since the disease often affects young patients, prosthetic arthroplasties rarely last for their lifetimes and usually necessitate multiple corrective procedures [2]. Therefore, joint-preserving treatment should be considered at the early stage to relieve pain and prevent progression.

Core decompression of the femoral head is a well-established joint-preserving procedure proposed to treat early-stage ONFH. However, a single large core tract has the risk of weakening subchondral bone support, which leads to a further collapse of the femoral head [3]. To overcome the limitations of this surgery, the multiple small-diameter drilling technique has been introduced and can provide the same benefit as core decompression [4]. In theory, this technique is a minimally invasive intervention that partly removes necrotic bone, provides mechanical support, and does not change the anatomic structure of the femoral head [3, 5–10]. Prior studies have demonstrated improvements in clinical symptoms after multiple drilling, failure of multiple drilling procedures is a main concern in patients and surgeons. However, due to homogenous study populations, determination of failure, sample sizes and follow-up duration, the efficacy of multiple drilling procedures in reducing the rate of conversion to THA remains controversial. Although there appears to be a consensus that core decompression or multiple drilling is more effective than nonoperative management for early-stage ONFH, the meta-analysis and review are on the basis of a few older small-scale randomized studies with short-term follow-up, which only provide limited quality of evidence [11, 12]. To our knowledge, there is few studies evaluating the efficacy of multiple drilling regimens in conversion to THA by comparing early-stage ONFH patients undergoing multiple drilling with the natural course.

Therefore, the primary purpose of the study was to compare the rate of conversion to THA in early-stage ONFH patients undergoing multiple drilling to that among patients undergoing a natural course. Furthermore, we identified risk factors for failure in a relatively large cohort of patients undergoing multiple drilling. The need for THA was the primary end point for defining failure. Additionally, we analyzed whether autogenous bone grafting is a protective factor against the need for THA. We hypothesized that there was significant difference in the rates of THA conversion in early-stage ONFH patients in the multiple drilling and the natural course group.

## Methods

### Patients

After approval from the institutional Clinical Trials and Biomedical Ethics Committee was obtained and the trial was registered at ClinicalTrials.gov, patients with early-stage nontraumatic ONFH were identified for eligibility, and all patients provided their written informed consent. We performed a retrospective, single-center, cohort study that enrolled early-stage nontraumatic ONFH from January 2008 to December 2018. The early-stage ONFH was defined as patients with modified Ficat and Arlet I and IIA/B stage [13]. The inclusion criteria included the following: (1) patients aged > 18 and < 65 years with nontraumatic ONFH, (2) patients who simultaneously underwent multiple drilling at the hip with early-stage osteonecrosis and THA at the contralateral side, (3) hips classification per the Ficat staging criteria as I and IIA/B stage according to modified Ficat and Alert classification for ONFH determined by preoperative X-ray and magnetic resonance (MR) imaging, and (4) only autogenous bone grafting, for patients who underwent bone grafting. Patients were excluded if they had or received any of the following: (1) pharmacological agents, including bisphosphonates, iloprost, enoxaparin, and statins; (2) biophysical therapies, including corporeal shockwave therapy and pulsed electromagnetic field therapy; (3) ankylosing spondylitis, osteoarthritis, and sickle cell disease; (4) femoral neck fracture (FNF) or femoral intertrochanteric fracture (FIF) during the period of follow-up; or (5) death, incomplete clinical or radiological data, and loss to follow-up. The indication for multiple drilling were restricted primarily to modified Ficat and Arlet stage I and IIA/B. All demographic data, including age, sex, mean body mass index (BMI), clinical and radiographic evaluations, intraoperative findings, and interventions, were collected. The search included all patients operated on by any of the 4 senior surgeons specializing in total joint arthroplasty in our unit.

**Fig. 1:**
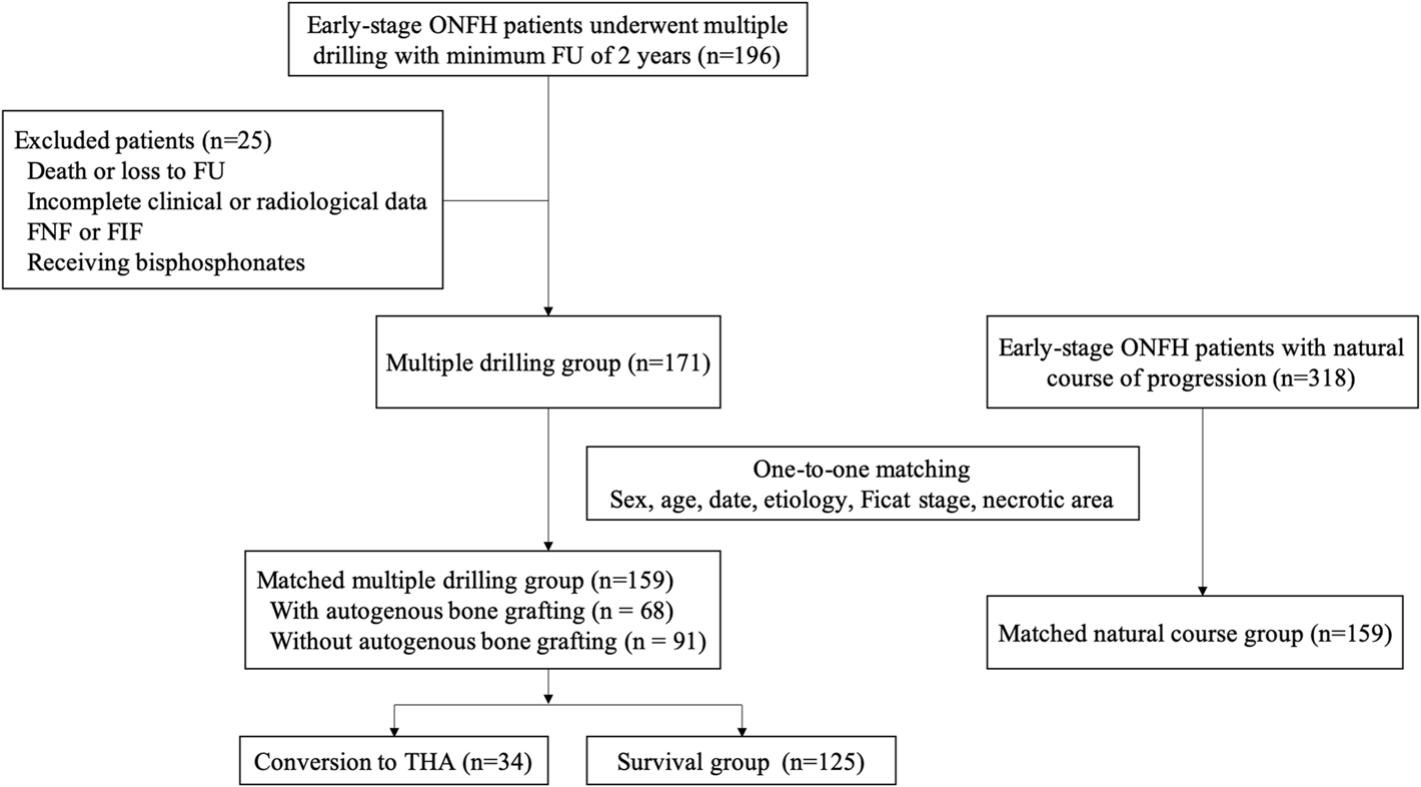
Flow diagram of patient enrollment. FU, follow-up; FNF, femoral neck fracture; FIF, femoral intertrochanteric fracture

We identified 196 patients diagnosed with early-stage ONFH who underwent multiple drilling between January 2008 and December 2018 and were followed for a minimum of 2 years. Of these, one patient was excluded due to death, eight patients were excluded due to loss to follow-up, six patients were excluded due to incomplete clinical or radiological data, eight patients were excluded due to FNF or FIF, and two patients were excluded due to receiving bisphosphonates. We also identified 318 patients who underwent THA and were diagnosed with early-stage ONFH at the contralateral hip that had a natural course of progression during the same follow-up period and included them, as the control group. Patients with a natural course of progression was defined as patients did not receive a therapeutic intervention for the purpose of treating or preventing osteonecrosis of the affected femoral head, including operative treatment, physical therapy, and biophysical stimulation. Patients with a natural course and patients who underwent multiple drilling were matched for sex, age at surgery (up to ±10 years), date of surgery (up to ±12 months), etiology, modified Ficat and arlet stage, and extent of necrotic lesion at a 1:1 ratio. The diagnosis and classification of ONFH was first defined by the ICD code and then confirmed by an orthopedic specialist based on their radiographic images. The final cohort consisted of 159 consecutive patients with a complete clinical and radiological follow-up for analysis, including 138 men (86.8%) and 21 women. Of note, as the indication for conversion to THA in so variable among surgeons, indication for conversion to THA in the current study was restricted to a new collapse of greater than 2 mm on plain radiographs for nontraumatic reason or onset of hip pain with a Harris hip score (HHS) less than 75 [14]. Radiographic progression was defined as a new collapse of greater than 2 mm (Ficat III stage) with or without decreased joint space. At the time of the latest follow-up, a total of 34 hips (21.4%) had been converted to THA because of onset of symptoms and collapse of the femoral head. These hips were compared with the subgroup to 125 hips (78.6%) following multiple drilling that had not been converted to THA, as shown in Fig. 1.

The surgical procedure of multiple drilling was performed as described by Mont et al. [15]. Multiple drilling was performed with patients under general anesthesia and in the supine position. The affected hip was rotated internally at 15°. A straight incision was created from 1.5 cm under the tip of the greater trochanter. A 3.0- to 5.5-mm guide pin was inserted into the lateral cortex of the femur toward the necrotic lesions from proximal to distal. After insertion into 10- to 12-mm pieces, the alignment of the guide pin was checked under fluoroscopy guidance to ensure that the pin was in the correct direction. The pin was advanced through the femoral neck into the femoral head at the appropriate depth. Similarly, another one or two additional parallel pins were inserted toward the necrotic lesions. Next, drill channels were created using a cannulated drill bit until it reached 5 mm beneath the subchondral bone. For 66 multiple drilling patients who received autogenous bone grafting, cancellous bone harvested from the contralateral femoral head was split into multiple pieces and packed into small holes with hammer blowing.

### Assessments

**Fig. 2:**
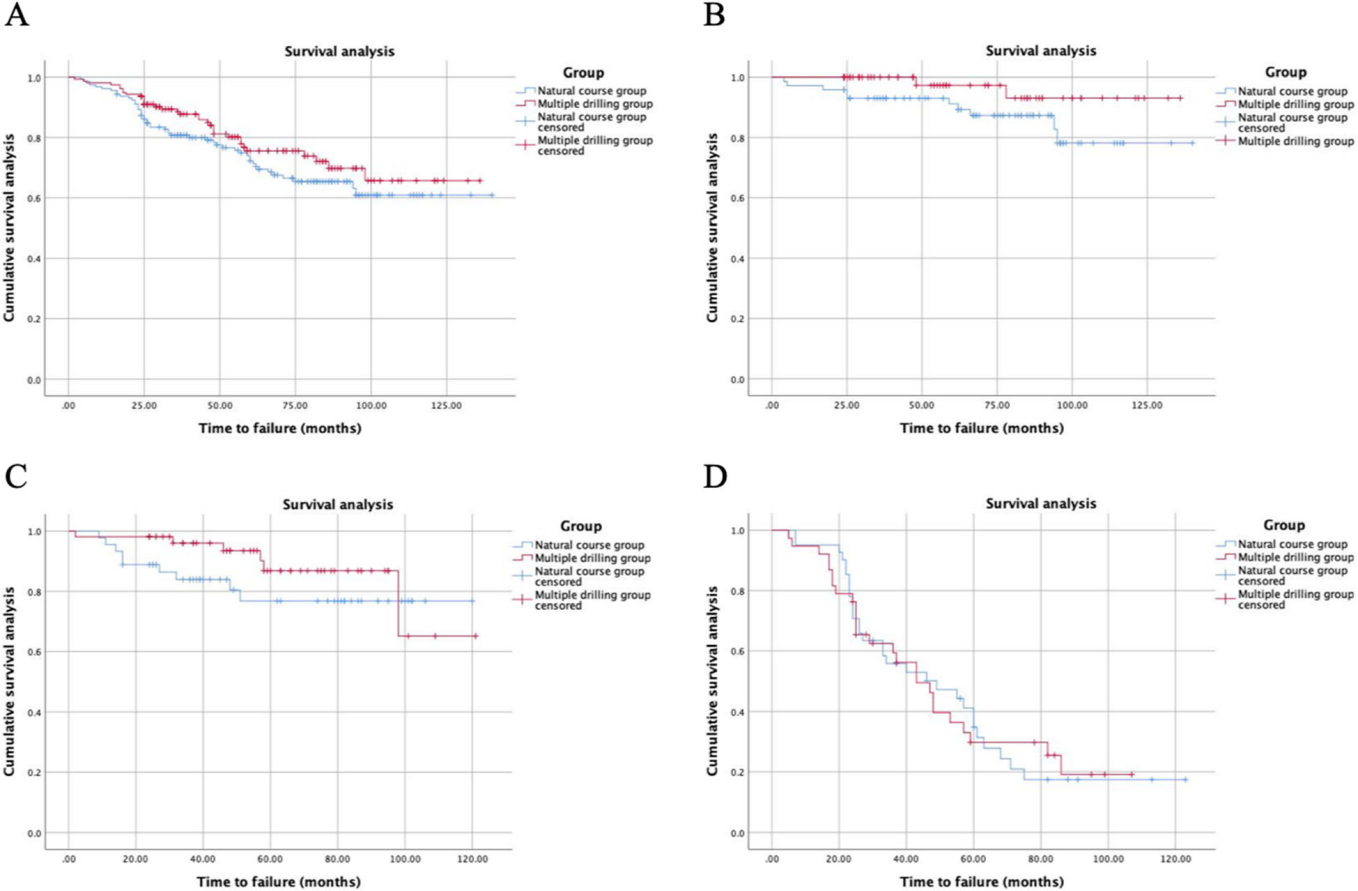
Radiographs of a patient in the multiple drilling group (5 l years old, male, and corticosteroid induced). **A** X-ray, **B** T2 image on transverse position, **C** T1 image on transverse position, and (**D**) T2 image on midcoronal position before surgery showed that the right femoral head had collapsed with osteoarthritis and that the left femoral head had cystic degeneration. The percentage extent of necrotic lesion was categorized to 15–30%. **E** Patients underwent simultaneous THA and multiple drilling. Forty-nine months after multiple drilling procedures, the left femoral head collapsed (**F**), and patients received THA in the left hip (**G**). Postoperative radiographic view (**H**) at the 4-year follow-up after left THA demonstrated that the acetabular and femoral components were stable

The etiologies, with or without preoperative symptoms, the preoperative HHS, and the postoperative work intensity [16] were recorded. Radiographic images were evaluated by two independent observers, who were blinded to whether the patient underwent multiple drilling, autogenous bone grafting, and conversion to THA. These included X-ray parameters (postoperative alpha angles, superior and medial joint space, radiographic parameters of hip dysplasia, and radiographic parameters of CAM-type femoroacetabular impingement (FAI)) and MR imaging parameters (location of necrotic lesion, extent of necrotic lesion, and bone marrow edema (BME)). Lesion location was assessed on midcoronal MR imaging using a system described by Sugano et al. [17]. The extent of the necrotic lesion was measured according to the method described by Steinberg et al. (mild, < 15%; moderate, 15–30%; severe, > 30%) [18], as shown in Fig. 2. The process of measurement involved outlining the necrotic lesion and the entire femoral head on both the anteroposterior and lateral MRI, determining the percentage of necrotic lesion in each of views separately. This method has been proved to be more accurate and reasonably reliable compared with other angular measurements [19].

### Statistical analysis

First, the distribution of data was tested by Kolmogorov-Smirnov’s test. Continuous variables were analyzed with the independent-sample t-test for normally distributed data or the Mann-Whitney U test for skewness distribution data, while categorical variables were compared using Fisher’s exact test. The primary analyses were to evaluate the influence of multiple drilling by comparing the rates of conversion to THA in patients following multiple drilling and patients with a natural course of progression by Kaplan-Meier curves and log-rank tests. Next, we created univariate and multivariate Cox proportional hazards to identify independent risk factors that affect survival rates in the multiple drilling group while adjusting for other covariates. Covariates with a *P* value < .05 by univariate analysis were integrated into the multivariate regression model. Finally, we analyzed the potential influence of autogenous bone grafting on the failure rate using Kaplan-Meier and log-rank tests. All analyses were carried out using SPSS Version 26 (IBM Corporation, Armonk, NY, USA). For this study, the level of statistical significance was set at *P* < .05.

## Results

### Comparison between patients with a natural course and who underwent multiple drilling

**Fig. 3:**
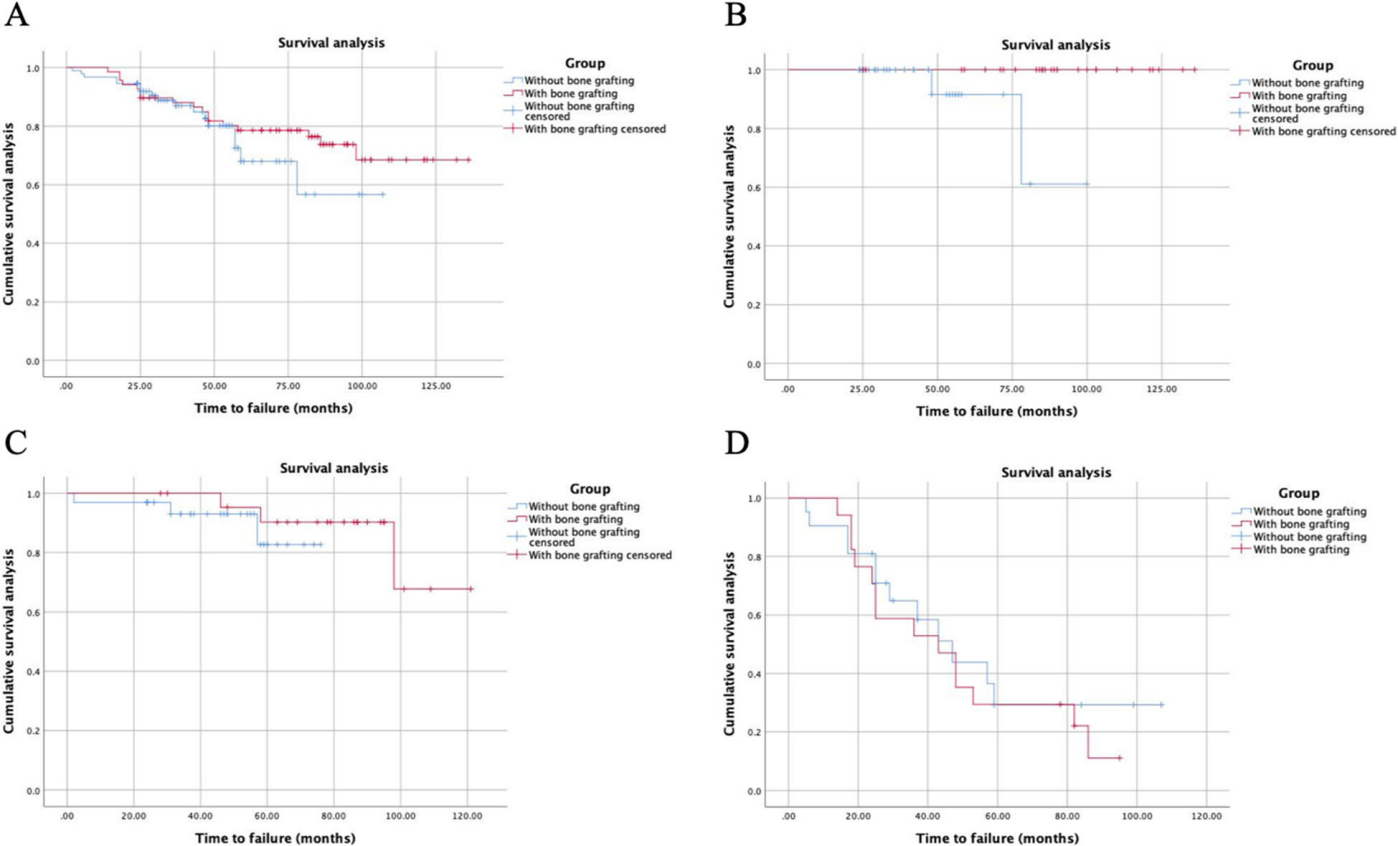
**A** Survival analysis of conversion to THA between patients who underwent multiple drilling and patients with a natural course of osteonecrosis progression. The difference was not statistically significant. (Kaplan-Meier method with long-rank test *P* = .191). Depending on the different extent of necrotic lesion, patients receiving multiple drilling showed near significant difference on survival rate in patients with necrotic lesion < 15% (**B**). However, neither extent of necrotic lesion of 15–30% (**C**) nor necrotic areas > 30% (**D**) with multiple drilling had an influence on conversion to THA compared to patients with natural course (*P =* .227, .995, respectively)

We matched the multiple drilling patients to 159 patients with a natural course of progression. Table 1 shows and compares demographic characteristics and radiological measurements between the 2 groups. The mean follow-up time (standard deviation) was 58.5 months (31.0) for all patients, 56.2 (30.2) months for the multiple drilling group and 60.8 (31.7) months for the natural course group (*P* = .191). In the multiple drilling group, the survival rates were 98.1% (95% confidence interval 95.9–99.9%) at 1 year, 75.6% (95% confidence interval 67.8–83.4%) at 5 years and 65.7% (95% confidence interval 53.7–77.7%) at 10 years, and they were 96.2% (95% confidence interval 93.3–99.1%), 72.2% (95% confidence interval 64.8–79.6%), and 60.9% (95% confidence interval 51.1–70.7%), respectively, in the natural course group (log-rank, *P* = .191). A total of 32 patients (20.1%) in multiple drilling group and 27 patients (17.0%) in natural course group had radiographic progression, and a total of 34 patients (21.4%) in multiple drilling group and 30 patients (18.9%) in natural course group went on to THA within the follow-up. Among patients with extent of necrotic lesion less than 15%, patients receiving multiple drilling (*n* = 66) had near significant influence on survival rate compared to patients (*n* = 73) with natural course (*P =* .069). However, neither extent of necrotic lesion of 15–30% nor necrotic lesion > 30% with multiple drilling had an influence on conversion to THA compared to patients with natural course (*P =* .227, .995, respectively), as shown in Fig. 3.

**Table 1:** Baseline characteristics of patients

Variable	Failure group	Survival group	*P* value	Multiple drilling group	Control group	*P* value
	(*n* = 34)	(*n* = 125)		(*n* = 159)	(*n* = 159)	
Age at surgery (y)	42.7 ± 7.8	43.2 ± 8.5	.767^a^	43.1 ± 8.4	44.5 ± 8.5	.131^a^
Gender (male/female)	30/4	108/17	1.000^b^	138/21	138/21	1.000^b^
Body mass index (kg/m^2^)	24.9 ± 4.8	23.9 ± 3.6	.178^a^	24.2 ± 3.9	23.8 ± 2.9	.307^a^
Etiology			.201^b^			1.000^b^
Alcohol abuse	24 (70.6%)	80 (64.0%)		104 (65.4%)	104 (65.4%)	
Corticosteroid use	7 (20.6%)	18 (14.4%)		25 (15.7%)	25 (15.7%)	
Idiopathic	3 (8.8%)	27 (21.6%)		30 (18.9%)	30 (18.9%)	
Modified Ficat and Arlet stage			<.001^b^			1.000^b^
Stage I	2 (5.9%)	14 (11.2%)		16 (10.1%)	16 (10.1%)	
Stage II A	10 (29.4%)	92 (73.6%)		102 (64.2%)	102 (64.2%)	
Stage II B	22 (64.7%)	19 (15.2%)		41 (25.8%)	41 (25.8%)	
Asymptomatic/sypmptomatic	23/11	77/48	.555^b^	100/59	93/66	.491^b^
Preoperative Harris score	88.2 ± 4.9	87.4 ± 5.1	.395^a^	87.6 ± 5.1	87.5 ± 8.1	.947^a^
Necrotic location			<.001^b^			.235^b^
A	2 (5.9%)	20 (16.0%)		22 (13.8%)	32 (20.1%)	
B	1 (2.9%)	45 (36.0%)		46 (28.9%)	34 (21.4%)	
C1	12 (35.3%)	47 (37.6%)		59 (37.1%)	55(34.6%)	
C2	19 (55.9%)	13 (10.4%)		32 (20.1%)	38 (23.9%)	
Extent of necrotic lesion			<.001^b^			1.000^b^
< 15%	2 (5.9%)	64 (51.2%)		66 (41.5%)	66 (41.5%)	
15–30%	6 (17.6%)	49 (39.2%)		55 (34.6%)	55 (34.6%)	
> 30%	26 (76.5%)	12 (9.6%)		38 (23.9%)	38 (23.9%)	
Bone marrow edema (no. of hips)	15 (44.1%)	13 (10.4%)	<.001^b^	27 (17.0%)	20 (12.6%)	.343^b^
Alpha angle	47.8 ± 7.1	48.2 ± 7.2	.825^a^	48.1 ± 7.1	50.1 ± 6.9	.009^a^
CAM type FAI (no. of hips)	3 (8.8%)	14 (11.2%)	1.000^b^	17 (10.7%)	25 (15.7%)	.246^b^
Hip dysplasia (no. of hips)	3 (8.8%)	13 (10.4%)	1.000^b^	16 (10.1%)	12 (7.5%)	.553^b^
Medial joint space (mm)	9.1 ± 2.2	9.4 ± 2.0	.355^a^	9.3 ± 2.2	9.5 ± 2.2	.400^a^
Superior joint space (mm)	4.6 ± 0.9	4.6 ± 1.1	.853^a^	4.6 ± 1.0	4.5 ± 1.0	.668^a^
Postoperative Work intensity			<.001^b^			.164^b^
Grade 0–1	14 (41.2%)	109 (87.2%)		123 (77.4%)	111 (69.8%)	
Grade 2	12 (35.3%)	10 (8.0%)		22 (13.8%)	35 (22.0%)	
Grade 3–4	8 (23.5%)	6 (4.8%)		14 (8.8%)	13 (8.2%)	
Bone grafting (no. of hips)	17 (50.0%)	51 (32.1%)	.435^b^			
Failure (no. of hips)				34 (21.4%)	49 (30.8%)	.191^c^

*FAI* femoroacetabular impingement

^a^ Student *t* test

^b^ Fisher’s exact test

^c^ Log-rank text

### Risk factors for failure after multiple drilling

After adjustment for covariates using the Cox proportional hazard model, the extent of necrotic lesion, the presence of BME on MR imaging, and postoperative work intensity were associated with a higher rate of conversion to THA after multiple drilling procedures (*P* = .001, *P* = .046, and *P* = .001). The results of the binary regression analysis are summarized in Table 2.

**Table 2:** Univariate and multivariate Cox regression analysis of association between different factors and failure of core decompression

Coefficient	Univariate analysis			Multivariate analysis		
	Hazard Ratio	*P* value	95% CI	Hazard Ratio	*P* value	95% CI
Ficat stage						
Stage I	1 (reference)	<.001	1 (reference)	1 (reference)	.641	1 (reference)
Stage II A	.781	.749	.171–3.565	1.741	.491	.359–8.432
Stage II B	4.749	.035	1.115–20.221	1.162	.849	.249–5.413
Preoperative Harris score	1.051	.190	.976–1.132	–	–	–
Necrotic location						
A	1 (reference)	<.001	1 (reference)	1 (reference)	.582	1 (reference)
B	.418	.538	.026–6.698	.306	.411	.018–5.163
C1	5.242	.111	.685–40.097	1.231	.857	.127–11.896
C2	18.644	.004	2.494–139.395	1.458	.752	.140–15.145
Extent of necrotic lesion						
< 15%	1 (reference)	<.001	1 (reference)	1 (reference)	.001	1 (reference)
15–30%	3.708	.109	.747–18.408	3.170	.196	.551–18.255
> 30%	32.521	<.001	7.695–137.444	22.293	.001	2.506–141.752
Bone marrow edema	3.814	<.001	1.935–7.518	2.188	.046	1.015–4.719
Postoperative work intensity						
Grade 0–1	1 (reference)	<.001	1 (reference)	1 (reference)	.001	1 (reference)
Grade 2	6.004	<.001	2.768–13.022	3.579	.002	1.583–8.092
Grade 3–4	6.771	<.001	2.833–16.186	5.036	.001	1.863–13.615

Variables included in the multivariate model if univariate *P* value <.05

### Influence of autogenous bone grafting in multiple drilling patients

**Fig. 4:**
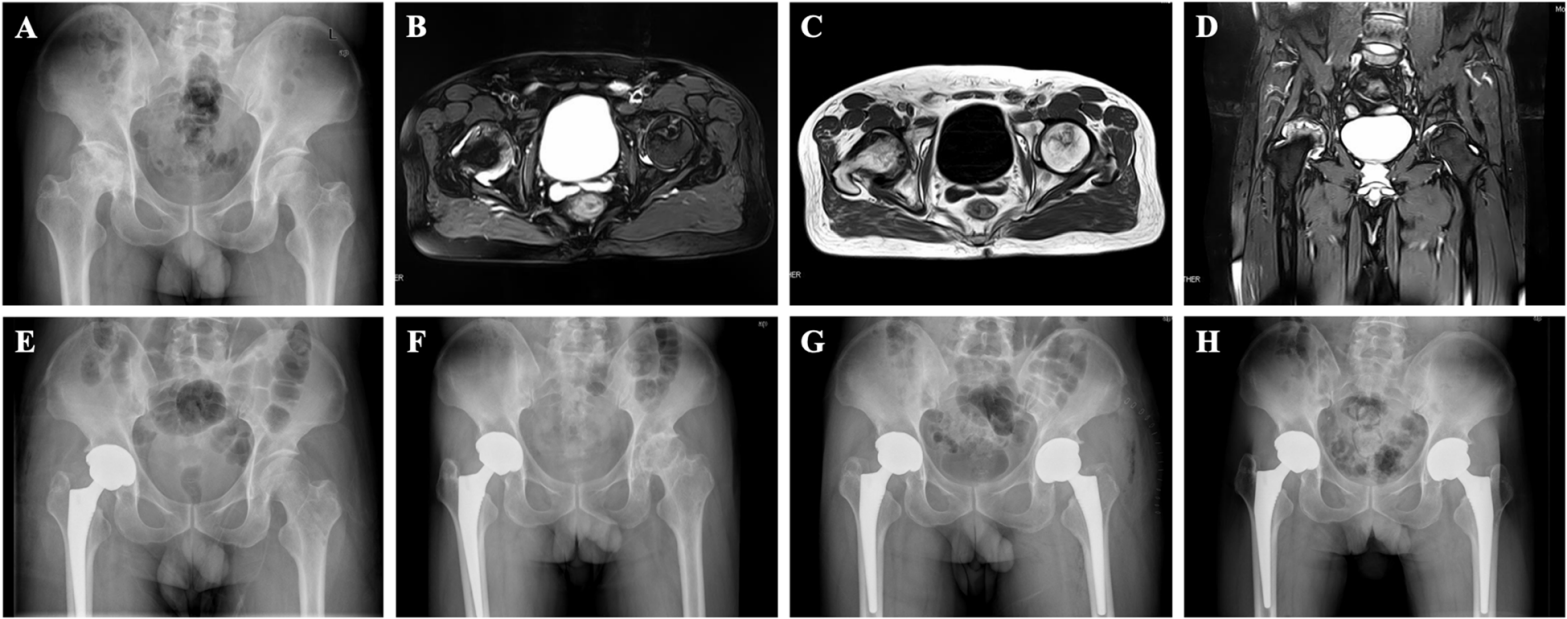
**A** Survival analysis of conversion to THA between patients who underwent multiple drilling combined with and without autogenous bone grafting (*P* = .373, Kaplan-Meier method with log-rank test). Depending on the different extent of necrotic lesion in ONFH patients who underwent multiple drilling, **B** the survival analysis showed a significant difference in patients with necrotic lesion < 15% (*P* = .007) and no significant difference in patients with necrotic lesion of 15–30% (**C**) or > 30% (**D**) (*P* = .453 and .657, respectively)

In the Kaplan-Meier analyses, autogenous bone grafting (*n* = 68) in multiple drilling patients had no influence on the survival rate compared to patients (*n* = 91) with multiple drilling alone (*P =* .373). However, multiple drilling patients with extent of necrotic lesion less than 15% who received autogenous bone grafting (*n* = 28) had a significantly lower risk of conversion to THA than patients (*n* = 38) receiving multiple drilling alone (*P =* .007). Among patients with necrotic lesion of 15–30%, patients who received autogenous bone grafting (*n* = 23) had no influence on survival rate compared to patients (*n* = 32) receiving multiple drilling alone (*P =* .453). Among patients with the extent of necrotic lesion > 30%, patients who received autogenous bone grafting (*n* = 17) also had no influence on survival rate compared to patients (*n* = 21) receiving multiple drilling alone (*P =* .657), as shown in Fig. 4.

## Discussion

In this retrospective study, there was no significant difference in the rate of THA conversion between the multiple drilling group and the natural course group. A larger extent of necrotic lesion, the presence of BME on MR imaging, and a higher postoperative work intensity were identified as independent risk factors for conversion to THA in patients following multiple drilling procedures for the treatment of early-stage osteonecrosis. Autogenous bone grafting had a protective effect on this risk only in patients with extent of necrotic lesion less than 15%.

Most orthopedic surgeons agree that treatment measures should be taken to slow progression and to prevent THA in early-stage ONFH [4]. Multiple drilling is one of the most widespread joint-preserving procedures proposed in recent decades [3]. In theory, this technique is a minimally invasive intervention that provides mechanical support and does not change the anatomic structure of the femoral head which may avoid facture or collapse of the femoral head. However, depending on different types of augmentations, the sample size, and the period of follow-up, variable rates of failure have been reported [3, 5, 6, 8, 20, 21], and the efficacy of multiple drilling remains controversial. Although several authors have reported improvements in clinical symptoms [3, 6, 8], failure of multiple drilling procedures is a main concern in patients and surgeons. In the current study, there was no difference in the rate of THA conversion in patients following multiple drilling procedures and with a natural course. Similar to our study, a network meta-analysis including only randomized controlled trials demonstrated no differences in the rates of THA conversion and radiologic progression across all core decompression modalities and nonoperative treatment [22]. Another meta-analysis also indicated that multiple drilling did not have significantly better effect on the prevention of THA progression than other therapeutic interventions [23]. The rate of survival following multiple drilling (78.3%) in our cohort appears to be higher than that reported by the majority of previous studies [6, 24]. The possible reason may be that the present study included only ONFH hips with modified Ficat and Arlet stage I and II. The survival rate of patients with early-stage ONFH was significantly higher than that of patients with postcollapse ONFH [6, 25].

There is constant discussion about the effectiveness of multiple drilling combined with autogenous bone grafting [3]. Autogenous bone grafting is sometimes performed as an ancillary procedure for multiple drilling that may provide structural support for new bone formation with mild inflammatory reactions. Previous studies reported that the success rate of autogenous bone grafting ranged from 55 to 87% at a follow-up period of 2 to 9 years postoperatively [26–31]. Nelson et al. [32] reported that 52 hips treated by bone grafting had a high rate of radiological progression and concluded that the technique was not effective in halting progression. Survival rates in our cohorts were significantly higher only in patients with small extent of necrotic lesion less than 15%, while autogenous bone grafting did not have a protective effect on the risk of conversion to THA in all early-stage ONFH patients. This is a new finding compared to prior published studies on this matter [25–30]. It may be that autogenous bone provides only short-term structural support after grafting. For patients with intermediate to larger necrotic lesion, necrotic lesion healing and subchondral bone remodeling may be difficult. Another possible reason may be that the cancellous bone graft in our cohort was harvested from the contralateral femoral neck. Previous studies demonstrated that decreased activity of bone marrow cells in the intertrochanteric region and iliac crest may provide insufficient osteoblasts to meet the need for bone remodeling in the early stage of osteonecrosis [33]. Although this technique has numerous theoretical advantages for the treatment of early-stage lesions, high-quality randomized trials are needed to confirm its efficacy.

A larger extent of necrotic lesion was found to be associated with a higher rate of conversion to THA in the present study. This finding was in line with the results of previous studies [6, 34]. In a review by Jay R. et al. [34], lower failure rates were found in patients with necrotic lesions involving less than 15% of the femoral head or with a necrotic angle of less than 200°. Won Seok et al. reported similar results: the survival rates of small necrotic lesions (100%) and medium-sized lesions (84.1%) were significantly higher than those of large necrotic lesions (53.8%) [6]. Stefan et al. reported that no treatment failure was observed in patients with remaining necrosis of less than 1000 mm^3^ at a mean follow-up of 33 months [35]. One reason for this association may be intuitive as patients with larger necrotic lesion in subchondral lesions may have less mechanical support of the femoral head and a higher risk of hip collapse. Another explanation could be that the amount of remaining necrotic tissue in the larger necrotic lesion was still substantial after small diameters of drilling. Postoperative magnetic resonance imaging showed that removal of the necrotic tissue was rarely complete [36].

Another factor associated with higher rates of conversion to THA was a higher postoperative work intensity. As the disease often affects young and active ONFH patients, the majority of patients wish to return to normal work and have a demand for a high level of physical function. This risk factor for conversion to THA after multiple drilling has not been mentioned in previous studies. In our cohort, 123 (77.3%) of 159 patients had a low level of work intensity after multiple drilling. However, the proportion of patients with medium and high work intensity was significantly higher in the failure group. A high level of work intensity, especially when carrying a heavy load, may increase the load on the hip, which results in accelerated hip collapse.

Interestingly, the presence of BME on MR imaging was identified as a risk factor for conversion to THA after multiple drilling in the current study. Previously it has been shown that BME of ONFH is highly correlated with the onset of hip pain and an increased risk of subchondral fracture [37, 38]. However, to date, there is no study evaluating the association of BME and conversion to THA after multiple drilling in early-stage ONFH. The underlying mechanisms of this identified link remains elusive, but a possible reason may be that BME represents a secondary sign of subchondral fracture of femoral head. Several prior studies have demonstrated that the presence of subchondral fracture in osteonecrosis is associated with the subsequent collapse of the femoral head which may result in conversion to THA [39, 40]. Another possible interpretation is that elevated intramedullary pressure caused by BME in the necrotic area may be considered a marker for progression to advanced osteonecrosis. Ito et al. [41] have demonstrated that the radiographic progression of ONFH patients with BME was significantly advanced than those without BME and concluded that the presence of BME might be a sign for deterioration of disease.

We studied the potential association between various radiographic parameters of FAI and failure of multiple drilling. Although the pathologies of the two diseases are totally different, previous studies have reported that the prevalence of CAM-type deformities is greatly increased in patients with ONFH and that CAM-type FAIs may act as a mechanical factor in developing ONFH [42, 43]. In contrast, the prevalence of FAI (10.7%) in our cohort was significantly lower than that in previous studies, and the association between FAI and failure of multiple drilling was not found. The possible reason may be that the present study included only early-stage osteonecrosis with integrity of the femoral head. For postcollapse ONFH, structural abnormalities of the head-neck junction may lead to impaired blood supply and reduced head-neck offset which accelerate the progression of disease.

We note that there are several limitations in the current study. First, we defined the failure of multiple drilling as conversion to THA. Poor functional outcome and radiographic progression of disease were not included as indicators of failure in our study. This design was deliberate. Absolute failure of multiple drilling, namely, conversion to THA, is an undisputed indicator of failure. With different standards of functional and radiographic outcomes, it is difficult to define the issue of functional and radiographic failure. Second, retrospective data decreased the level of evidence and inevitably resulted in some bias and inaccuracies. Third, there are 4 different surgeons performed multiple drilling procedures in our unit, which inevitably led to variables. However, each of the 4 surgeons had a high volume of ONFH every year, performed similar surgical techniques, and followed the same protocols in treating patients with ONFH.

## Conclusion

Our findings demonstrated that multiple drilling is not effective in delaying or preventing THA in early-stage ONFH patients compared with a natural course of progression. A larger extent of necrotic lesion, the presence of BME on MR imaging, and higher postoperative work intensity were identified as risk factors for treatment failure and conversion to THA in patients following multiple drilling. We emphasized the importance of evaluating necrotic lesion on MR imaging rather than stage alone, and hip-preservation surgeons should be aware of these risk factors for careful patient selection.

## Data Availability

The datasets used or analyzed in the current study are available from the corresponding author on reasonable request.

## Abbreviations

ONFH: Osteonecrosis of the femoral head
THA: Total hip arthroplasty
MR imaging: Magnetic resonance imaging
BMI: Body mass index
HHS: Harris hip score
FAI: Femoroacetabular impingement
FNF: Femoral neck fracture
FIF: Femoral intertrochanteric fracture
